# Active particle feedback control with a single-shot detection convolutional neural network

**DOI:** 10.1038/s41598-020-69055-2

**Published:** 2020-07-28

**Authors:** Martin Fränzl, Frank Cichos

**Affiliations:** Molecular Nanophotonics Group, Peter Debye Institute for Soft Matter Physics, Universität Leipzig, Linnéstr. 5, 04103 Leipzig, Germany

**Keywords:** Microscopy, Colloids, Computational methods

## Abstract

The real-time detection of objects in optical microscopy allows their direct manipulation, which has recently become a new tool for the control, e.g., of active particles. For larger heterogeneous ensembles of particles, detection techniques are required that can localize and classify different objects with strongly inhomogeneous optical contrast at video rate, which is often difficult to achieve with conventional algorithmic approaches. We present a convolutional neural network single-shot detector which is suitable for real-time applications in optical microscopy. The network is capable of localizing and classifying multiple microscopic objects at up to 100 frames per second in images as large as $416 \times 416$ pixels, even at very low signal-to-noise ratios. The detection scheme can be easily adapted and extended, e.g., to new particle classes and additional parameters as demonstrated for particle orientation. The developed framework is shown to control self-thermophoretic active particles in a heterogeneous ensemble selectively. Our approach will pave the way for new studies of collective behavior in active matter based on artificial interaction rules.

## Introduction

Optical microscopy can provide structural information but also allows us to follow dynamical processes from single molecules and nanoparticles to cells and organisms. Images with high spatial, temporal, and also spectral resolution may be obtained. Especially the ability to see dynamic processes opens the possibility to influence these processes in real-time via feedback control. In the field of single-molecule detection, this has been demonstrated with the electrokinetic or the thermophoretic trap^1–3^. In both cases, the optical images are analyzed in real-time to extract particle or molecule positions to control electric or temperature fields for positioning purposes. Similarly, feedback control can explore new physics in optical tweezers^4^ or control active particles by specific rules^5–7^. Such synthetic active particles mimic the active propulsion of biological species like bacteria or larger organisms^8^. While the biological species are able to exchange signals to form collective states like swarms or to break the action–reaction principle, their synthetic counterparts are still missing these features. Feedback control of active particles introduces the possibility to respond to external events or neighboring active particles by complex behavioral rules allowing a completely new approach to study the consequences of sensorial interactions in biological species. This field is of quickly growing interest^9–11^ yet the control is still focused on active particles of the same size and shape due to the lack of more advanced real-time image processing algorithms. New algorithms which can localize and classify a large amount of active species of different size, shape and signal-to-noise ratio and will pave the way to new studies. The main requirements for those new approaches are (1) to be able to process images at video rate, (2) the ability to differentiate between multiple species, (3) to work at different optical contrasts and signal-to-noise ratios (SNR). These requirements are often met by algorithmic approaches using thresholding and centroid calculation or even more advanced versions. Yet, the more complex the image is, e.g., having particles with different contrasts, the bigger is the computational effort that has to be spend at the cost of speed^12–14^. Recently, machine learning methods have been introduced to the field of optical microscopy and single-particle detection. Those methods are used for image segmentation, holographic reconstruction, or also particle tracking^15–20^. Methods for particle and object tracking currently employed in digital microscopy are based on convolutional neural networks designed for post-processing, i.e., they are optimized for accuracy, not speed. Their approach is limited by the fact that they often slide smaller regions of interest over a larger image to detect multiple objects of the same class. The network, therefore, has to be called multiple times per frame, which is time consuming. The detection of different object classes then even requires separate networks to be trained, which hampers their use for real-time detection considerably. Using neural networks which detect and classify objects in a single step would therefore be of considerable interest for the above mentioned feedback control applications.

**Figure 1:**
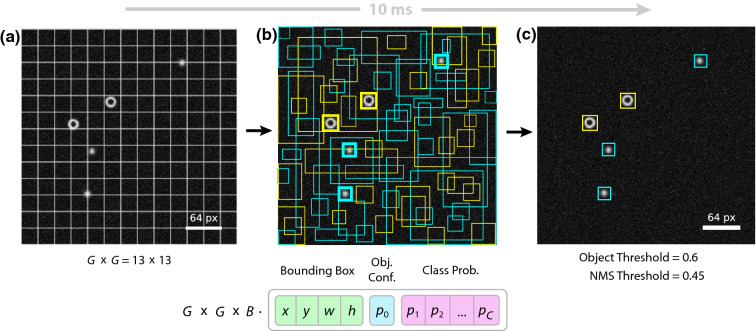
Detection principle. (**a**) The network takes an input RGB image of size $416 \times 416$ pixel and divides it into a $G \times G$ grid with $G = 13$. (**b**) For each grid cell it predicts *B* bounding boxes, confidence for those boxes and *C* class probabilities. Here, we use $B = 5$ and $C = 2$. These predictions are encoded in a $G \times G \times B \cdot (4 + 1 + C)$ output tensor. The line thickness of the bounding boxes in (**b**) depicts the object confidence whereas the color of the bounding box is selected according to the highest class probability. (**c**) Only bounding boxes with an object confidence larger than a certain object threshold are retained. A non-maximum suppression (NMS) algorithm and a NMS threshold value is used to remove overlapping bounding boxes that belong to the same object. Typical values are 0.6 and 0.45 for the object and NMS threshold, respectively.

We present a single-shot convolutional neural network enabling the detection and classification of objects in optical microscopy images in real-time. The network is employed in an optical microscopy setup for the manipulation of microparticles propelled by laser-induced self-thermophoresis. The actuation of these active particles is based on a feedback control of a steerable, focused laser beam and hence requires a detection of the positions of the particles as fast as possible. A single-shot neural network architecture^21,22^ together with a GPU implementation in LabVIEW allows us to perform the particle localization and classification in real-time at a speed of up to 100 frames per second (fps) for $416 \times 416$ pixel sized images where the processing speed is not limited by the number of objects available in the image. With the help of this approach we are able to actuate active particles in mixed samples with passive particles and at very low SNRs previously not accessible by algorithmic approaches.

## Results

### Network structure, training and deployment

The used single-shot detection approach is based on the TinyYOLOv2 network architecture^22^. It consists of 9 convolutional layers (Supplementary Section 1.1), where the first layers take an input RGB image of the size of $416 \times 416$ pixels. The input image is divided into $13 \times 13$ grid cells (Fig. 1a). For each grid cell the network predicts 5 bounding boxes. For each bounding box the position and size of an object as well as the confidence of detection and a probability for each class are predicted (Fig. 1b). Bounding boxes with confidence values above an object threshold are used for further evaluation (Fig. 1c). A non-maximum suppression (NMS) algorithm with a threshold value is used to remove overlapping bounding boxes that belong to the same object. The object class is assigned according to the maximum value of the predicted class probabilities. A more detailed description of the output decoding can be found in Supplementary Section 1.2.

The network is trained in Python/Keras using the TensorFlow backend^23–25^ on a GeForce GTX 1660 Ti GPU without any pre-trained weights. The corresponding Python scripts for the training of the network and the generation of synthetic training data are supplied with Supplementary Code 1 and explained in detail in Supplementary Sections 2–4. While the synthetic datasets used in this work resemble darkfield microscopy images of nano- and microparticles, Janus-type as well as rod-like and elliptical microparticles, any other training set may be used. Note that all images are assumed to be in focus without changing the contrast of diffraction patterns when defocusing. We train the network with a training set of 25,000 images and a validation set of 5,000 images for 10 epochs and a batch size of 8. The image generation takes about 30 min on an Intel Core i7 9700 K $8 \times 3.60\,\hbox {GHz}$ CPU and the training process about 1 h on a GeForce GTX 1660 Ti GPU.

The trained network graph is exported and deployed to a LabVIEW program developed in the lab which is controlling our microscopy setup. The LabVIEW implementation comprises dynamic link libraries (DLLs) written in C that take an RGB image as input and deliver the decoded output. To get the fastest possible image processing the DLLs are using the GPU supported TensorFlow C API. The details concerning the software can be found in Supplementary Code 1 and Supplementary Section 4. Using a GeForce GTX 1660 Ti GPU an inference time of about 10 ms is achieved for RGB images with a size of $416 \times 416$ pixels. This inference time might be further improved by employing a faster GPU or smaller input image sizes.

### Evaluation of the network performance

The performance of the network is evaluated for various synthetic datasets and is discussed in detail in Supplementary Section 6. We evaluate the accuracy of the position detection for single objects, close encounters, and the number of false/true positive and negative detections for multiple objects within an image. These parameters are evaluated for single and multiple class training data sets as a function of the SNR. The SNR of an image is defined as the ratio of the particles mean signal to the standard deviation of the signal. The details of the investigated datasets (Dataset 1–3) are provided in Supplementary Sections 3.1–3.3.

When trained with a single class dataset a root-mean-square error (RMSE) of the localization of about one pixel is obtained (Supplementary Figs. S16a, S17a). We achieve subpixel resolution using a class-dependent offset correction (see Supplementary Section 5 for details). For increasing SNR, the error decreases and saturates at a constant value of about 0.5 pixel after offset correction. This is contrary to algorithmic approaches, where the RMSE scales with the inverse of the SNR^26^. Thus, algorithmic approaches will yield better accuracy for high SNR for single class detection but also a stronger dependence on the SNR. For low SNRs in the range of 1–10 our network compares well to the localization accuracy of advanced, algorithmic methods^26^. While recent machine learning approaches have shown even better performance in terms of the localization accuracy^18^, their approach for multiple particle, multiple species detection is commonly more time consuming due to sliding window approaches, which require the network to be run multiple times for a single frame. This often precludes the application of these networks in situations where real-time information is required. For a two-class training dataset, the RMSE slightly increases and saturates for high SNRs at about 1 pixel (Supplementary Fig. S18a). For the identification of separate particles in close encounters Supplementary Figs. S16b and S17b illustrate that the predicted distance nicely reflects the true distance down to a value of $2\sigma$. Here, $2\sigma$ is the size of the particle. Remarkably, for ring-like particles, it is even possible to detect overlapping particles (Supplementary Fig. S17b). Furthermore, it is shown that even when one particle is by a factor of 10 darker than the other, the network still detects the two particles with the same accuracy as for equal contrast (Supplementary Figs. S16c, S17c). For more than two particles within an image, the detection performance is evaluated in terms of the percentage of true positive, false negative, and false positive detections (Supplementary Fig. S18c). The number of true positive detections starts to drop at $\mathrm {SNR} < 3$ at the cost of false negatives. At $\mathrm {SNR}= 1$, about 50% of the objects are still detected, while only about 1% are detected false positive. This is remarkable since at a SNR level of 1 it is even difficult to identify objects by eye (see Supplementary Fig. S16a for reference). Notably, no classification errors have been observed when tested on synthetic images.

**Figure 2:**
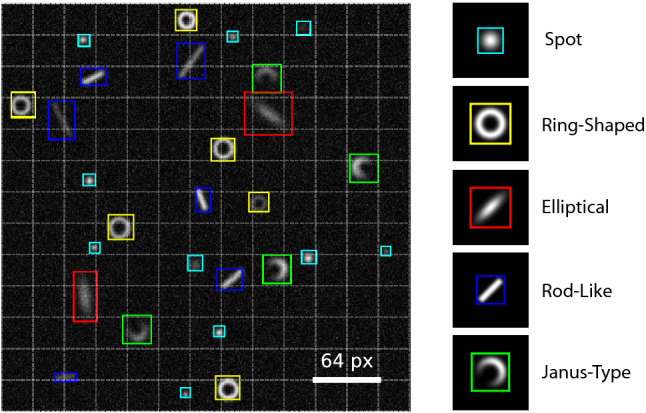
Multiple training classes. Predicted locations, sizes and classes for a test image for a model trained with five different particle classes.

The number of classes to be localized and classified in an image can be easily extended to more than two classes. In principle, the network can be trained for several thousand object classes^22^. Situations with multiple classes are very challenging for conventional algorithmic localization and classification^12–14,26–32^ even if the individual particles have a high SNR. As a demonstration, we have trained our network with a dataset containing five different particle classes: spots, ring-shaped and Janus-type particles as well as rod-like and elliptical particles (Dataset 5, Supplementary Section 3.5). Figure 2 illustrates the performance of the model. The different particle classes are accurately identified despite their different sizes, orientations, and intensities. Even rod-like particles are properly distinguished from elliptical particles. The latter two particles are very difficult to separate in algorithmic approaches. The proposed neural network-based detection technique will, therefore, be advantageous, especially in cases with multiple species and heterogeneous optical contrast.

**Figure 3:**
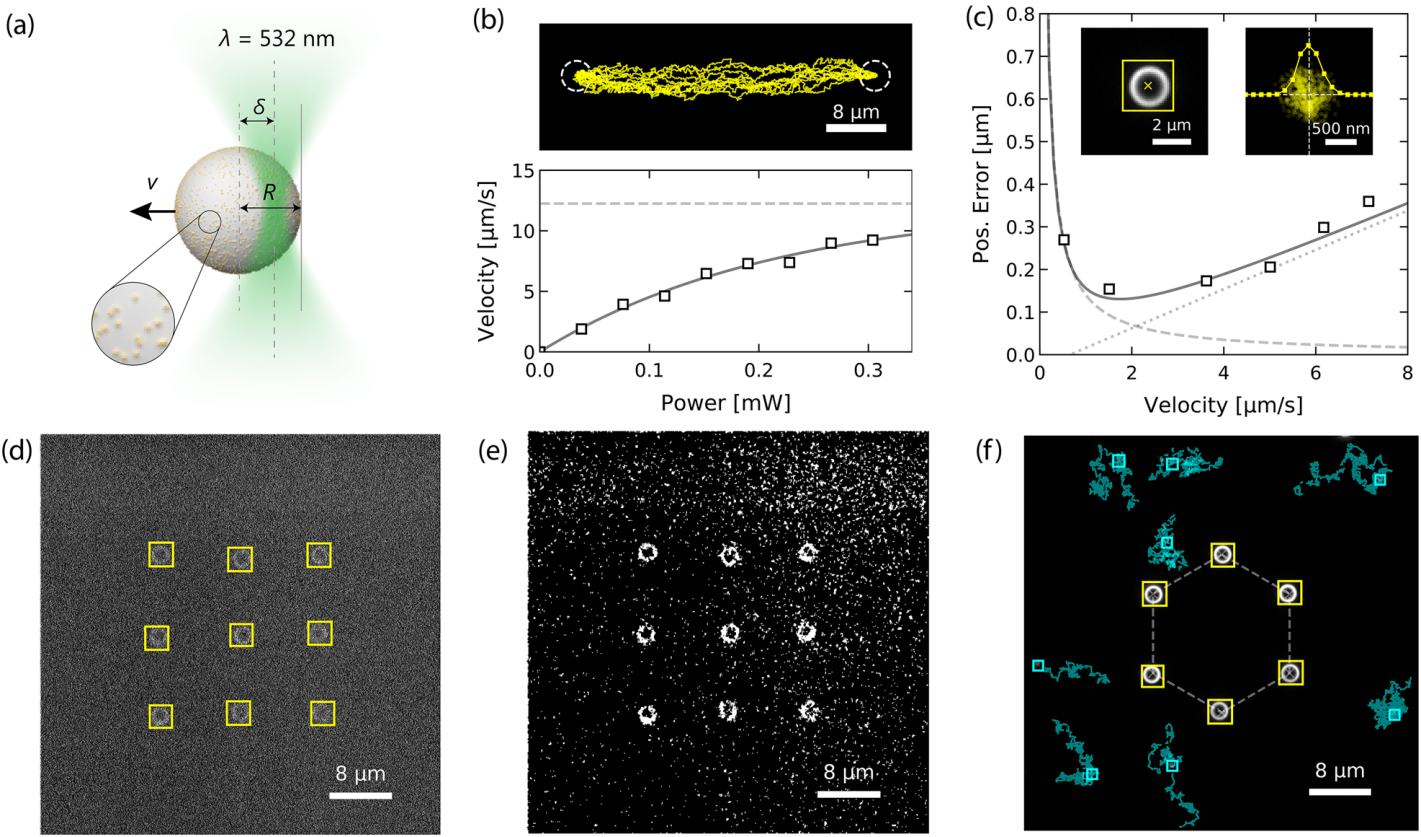
Experimental real-time detection for feedback optical microscopy. (**a**) Sketch of a self-thermophoretic active particle composed of a $2.2\,\upmu \hbox {m}$ diameter melamine formaldehyde (MF) particle ($R = 1.1\,\upmu \hbox {m}$) covered with 10 nm gold nanoparticles with a surface coverage of about 10%. When asymmetrically heated with a focused laser ($\lambda = 532~\hbox {nm}$) an inhomogeneous surface temperature is generated resulting in a self-thermophoretic motion away from the laser focus. The velocity of the particle ${v}_{\mathrm{th}}$ depends on the incident laser power and on the displacement of the laser focus $\delta$ from the particle center. The highest velocity is observed for $\delta \approx 0.5 R$. (**b**) The particle velocity derived from an experiment driving the particle between two target positions (Supplementary Video 1). With increasing laser power the particle velocity saturates at about $12\,\upmu \hbox {m/s}$ (dashed curve). The non-linear dependence (solid curve) is analyzed elsewhere^7^. The upper illustration shows the recorded trajectories for a power of 0.2 mW. (**c**) The control accuracy as function of the laser power derived from an experiment confining a single particle for a certain time at a target position (Supplementary Video 2). The dashed and dotted curves are the contributions from the 2D sedimentation (dashed) and the particle overshooting (dotted). The sum of both contributions is represented by the solid curve. The left inset depicts a sample detection and the right inset the 2D position distribution for a power of 0.1 mW. (**d**) Experimental feedback control of nine individual active particles in a grid pattern at low SNR (Supplementary Video 3). (**e**) Threshold representation of the image shown in (**d**) pre-processed with a $3\times 3$ median filter. (**f**) Experimental feedback control of six individual active particles in a hexagonal pattern (Supplementary Video 4) surrounded by passive $0.5\,\upmu \hbox {m}$ diameter polystyrene particles.

### Experimental feedback control of active particles

Considering the inference time of 10 ms and the localization RMSE of about 1 pixel as well as the independence of the processing speed on the number of particles in the image, the above-presented network is well suitable for real-time detection and feedback control of active particles. To demonstrate the capabilities of the network, we studied the feedback-controlled actuation of microparticles confined in a thin liquid film. The experimental test should also verify the model that has been trained with synthetic data under real experimental conditions, which may differ from the ideal training situation (reality gap). The particle suspension contains $2.2\,\upmu \hbox {m}$ diameter melamine formaldehyde (MF) particles as well as $0.5\,\upmu \hbox {m}$ diameter polystyrene (PS) particles. The surface of the MF particles is uniformly covered with gold nanoparticles of about 10 nm diameter with a surface coverage of about 10% (Fig. 3a). The particles are observed using darkfield illumination with an oil-immersion darkfield condenser and a $100\times$ oil-immersion objective. Details of the experimental setup and the sample preparation are available in Supplementary Sections 7 and 8. The MF particles appear with a ring-shaped intensity profile whereas the smaller PS particles have an approximately Gaussian intensity profile.

Illuminating the gold nanoparticles at the MF particle surface asymmetrically with a highly focused laser beam with a wavelength close to their plasmon resonance ($\lambda =532\, \mathrm{nm}$) generates an inhomogeneous surface temperature. This inhomogeneous surface temperature is resulting in a self-thermophoretic propulsion away from the laser focus (Fig. 3a)^6,7^. To control the active particle motion direction the laser focus needs to be placed at the circumference of the particle in real-time requiring the detection of the particle center position with sufficient accuracy and speed. This actuation scheme, which is similar to the photon nudging of Janus-type particles^5,6,33,34^, is achieved with the help of our neural network.

For the experimental detection the network was trained with a two-class dataset: Gaussian spots, as observed for the $0.5\,\upmu \hbox {m}$ PS particles and ring-shaped intensity profiles as observed for the $2.2\,\upmu \hbox {m}$ particles. Different magnifications are taken into account by training the network for different scales, e.g., different sizes of the two classes (Dataset 4, Supplementary Section 3.4). The laser in our setup is steered by an acousto-optic deflector. The camera was set to acquire images with a size of $512 \times 512$ pixels at an inverse frame rate of 40 ms (25 fps). To match the network input size the monochrome images from the camera are rescaled to $416 \times 416$ pixels and converted to grayscale RGB images. The inference of particle positions requires only about 10 ms. Additional 30 ms are needed for the processing of the particle positions and writing the uncompressed image data to disk.

We first evaluate the accuracy of the feedback control of a single active particle. To extract the dependence of the particle velocity on the laser power the particle is driven between two target positions (Fig. 3b, Supplementary Video 1). We find a non-linear scaling as the particle slips out of the laser focus during the time $\tau$ corresponding to the inverse frame rate. The origin of the nonlinearity is analyzed in detail elsewhere^7^. Here, we fit an empirical function $v = {v}_{\mathrm{max}} (1 - \exp (-b P))$, where *P* is the power and ${v}_{\mathrm{max}}$, *b* are fitting parameters (Fig. 3b, solid curve). A maximum velocity of ${v}_{\mathrm{max}} = 12\,\upmu \hbox {m/s}$ is found for $\tau = 40\,{\mathrm{ms}}$. The maximum velocity decreases exponentially with $\tau$, pointing out the significance of our network’s fast execution speed. To extract the dependence of the control accuracy on the heating power a single particle is confined at a target position for a certain time (Fig. 3c, Supplementary Video 2). We characterize the confinement by the positioning error $\sigma = \sqrt{\langle ({\mathbf{r}} - {\mathbf {r}}_{\mathrm{t}})^{2}\rangle }$, where ${\mathbf {r}}$ and ${\mathbf{r}}_{\mathrm{t}}$ are the coordinates of the particle and the target, respectively. The positioning error has two regimes. If the particle displacement during the inverse frame rate $\tau$ due to the self-thermophoretic propulsion is smaller than the displacement due to the diffusion, the positioning error is represented by a simple sedimentation model: the particle is radially driven towards the target with a velocity *v* against its diffusive motion with the diffusion coefficient *D*. In the steady state the characteristic length scale is $\sigma = \sqrt{6}D/v$ as indicated by the dashed curve in Fig. 3c. With increasing particle velocity the positioning error gets defined by an overshooting of the particle over the target position^7^. This is due to the finite sampling of the particle position with the inverse frame rate $\tau$. The overshooting distance is the traveled distance within the inverse frame rate $\tau$ and increases with increasing power as shown by the dotted curve in Fig. 3c. The sum of both contributions is $\sigma = \sqrt{6D^2/v^2 + v^2\tau ^2}$ depicted as solid curve in Fig. 3c. The minimum in the positioning error is found as ${\sigma}_{\mathrm{min}} = 140\,{\mathrm{nm}}$ for ${v}_{\mathrm{min}} = 1.8\,\upmu \hbox {m/s}$. To achieve a control accuracy as high as possible the inverse frame rate $\tau$ needs to be as small as possible, again, highlighting the demand for fast image processing in active particle feedback control.

To control multiple particles they are addressed by quickly multiplexing the laser focus with $\Delta t = 10\,\upmu {\mathrm{s}}$ between the different particle positions within the inverse frame rate time $\tau$. Therefore, the incident laser power is available for a time $\tau /N$ to each of the *N* particles decreasing the average laser power per particle. Figure 3d and Supplementary Video 3 demonstrate the control of nine individual active particles (yellow boxes) in a grid pattern at low SNR. Initially, the particles were randomly distributed in the field of view. When the feedback control is enabled each particle is driven towards its nearest target and eventually confined there. As the resulting arrangement is constantly actuated the structure is dynamic as can be seen from Supplementary Video 3. The structure can be maintained even at very low SNR were a real-time detection of the particles with algorithmic approaches is already quite challenging. Figure 3e shows a threshold representation of Fig. 3d, pre-processed with a $3\times 3$ media filter for reference. Figure 3f and Supplementary Video 4 demonstrate the control of six active particles (yellow boxes) with a background of passive PS particles (cyan boxes/trajectories). Here, in addition to Fig. 3d, a classification of the particles was required. Despite their lower intensity, the PS particles are still detected by the network and all particles are properly classified. Two additional control patterns are provided with Supplementary Videos 5 and 6. Notably, for all experiments, the same trained network (Dataset 4) was used and no fine-tuning of any parameters was required.

### Extension to additional parameters—orientation detection

**Figure 4:**
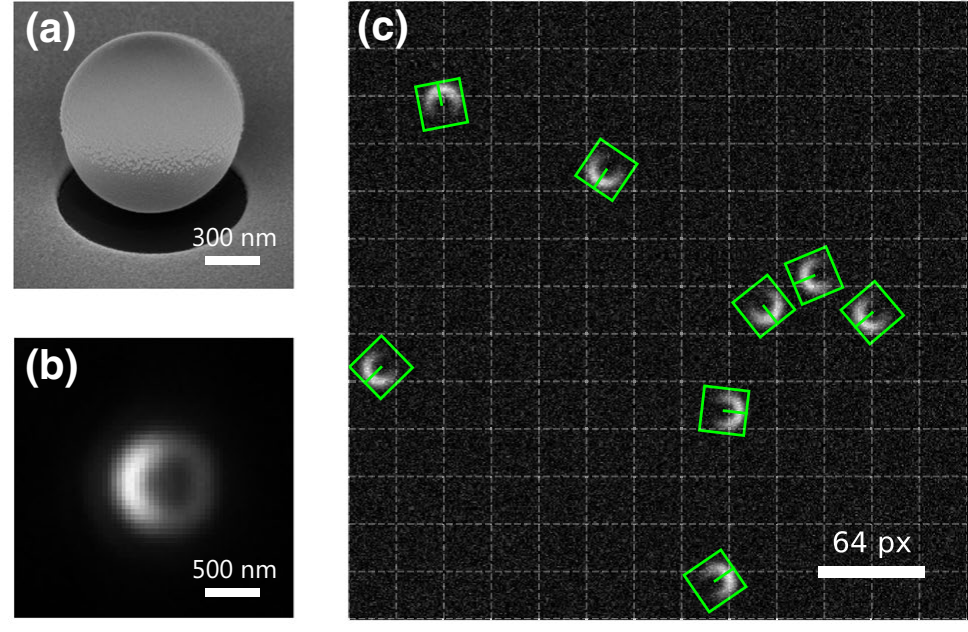
Extension of the network for orientation detection. (**a**) A scanning electron microscopy (SEM) image of a $1\,\upmu \mathrm {m}$ diameter Janus particle. (**b**) Darkfield image of a $1\,\upmu \mathrm {m}$ diameter Janus particle. (**c**) A sample image with synthetic Janus type particles and the predicted locations and orientations by the extended network.

While the discussion so far referred to particle positions and particle classes, one may extend the network also to include other parameters. The orientation of objects becomes particularly interesting when particles lack spherical symmetry or have anisotropic optical properties. As can be seen for the elliptical and rod-like particles in Fig. 2 the orientation of objects with a $180^{\circ }$ rotational invariance can be partly retrieved from the aspect ratio of the detected bounding boxes. Nevertheless, this yields an ambiguity of $90^{\circ }$ since one cannot distinguish between, e.g., $-45^{\circ }$ and $45^{\circ }$. In the case of objects with no rotational invariance and a quadratic bounding box, an orientation detection via the aspect ratio of bounding boxes is not possible at all. This is the case for Janus particles. Janus particles, when consisting of a hemispherical gold layer on top of a spherical polymer particle (Fig. 4a) result in moon-shaped darkfield images (Fig. 4b). The image, therefore, allows for detection of the particle orientation but not from the bounding box.

To allow also for the prediction of the orientation, the network needs to be modified and a new parameter, e.g., an angle $\varphi$, needs to be introduced to the loss function and the annotation format (Supplementary Section 9 and Code 2). Figure 4c shows the detection output of the extended network trained for Janus type particles^5,6,31^. An extension of the network to detect even more parameters such as the *z*-position of the particle or the out-of-plane rotation is easily possible emphasizing the flexibility of the network architecture^35,36^. A more detailed description of the extended network can be found in Supplementary Section 9.

## Summary

In summary, we have shown that the adaption of a single-shot convolutional neural network allows us to localize and classify objects in optical microscopy images with an inference time of about 10 ms. The speed of the classification and localization is independent of the number of particles and the complexity of the image. While algorithmic approaches will be faster and more accurate for simple images and particle shapes including one species and high signal-to-noise ratio (SNR > 10), our method is suitable for multiple species detection with large signal-to-noise variations in the image. We analyzed the network performance with synthetic images and demonstrated its experimental application to feedback actuated self-thermophoretic active particles while tracking individual gold nanoparticles in the background. Feedback controlled active particle systems deliver a unique possibility to create artificial interactions between synthetic active particles to mimic sensorial interactions in living species which break the action–reaction principle in physics. Using this approach we envision further applications in the control of active matter and a combination with other machine learning techniques, e.g., reinforcement learning for adaptive control and particle navigation^37^. The real-time localization and classification capability of the network presented here is an important step towards active particle systems that are fully self-regulated by machine learning techniques where the feedback control and image detection are interconnected. Autonomous systems have the potential to explore new emergent phenomena in heterogeneous active particle ensembles and might be even employed to discover spatial and temporal feedback policies that also drive group formation in living matter. Furthermore, our work has the potential to control processes in even more challenging imaging situations such as in biological species. The source code and scripts of our framework are open source and can be easily adapted and extended for these applications.

## Supplementary information

Supplementary Information 1.

Supplementary Video 1.

Supplementary Video 2.

Supplementary Video 3.

Supplementary Video 4.

Supplementary Video 5.

Supplementary Video 6.
