# Supplementary material for: Active particle feedback control with a single-shot detection convolutional neural network
Source: Sci Rep. 2020 Jul 28;10:12571. doi: 10.1038/s41598-020-69055-2 (PMC7387478; doi:10.1038/s41598-020-69055-2)
Supplement: Supplementary file 1 — Supplementary Information 1. [file 41598_2020_69055_MOESM1_ESM.pdf]

Supplementary Information

Active particle feedback control with a single-shot detection convolutional neural network

Martin Fränzl[1] and Frank Cichos[1,*]

[1] *Peter Debye Institute for Soft Matter Physics, Molecular Nanophotonics Group, Universität Leipzig, Linnéstr. 5, 04103 Leipzig, Germany.*

[*] *cichos@physik.uni-leipzig.de*

Contents

1 Neural Network Design **2**
1.1 Architecture 2
1.2 Output Decoding 3

2 Neural Network Training **4**
2.1 Loss Function 4
2.2 Synthetic Image Generation 6

3 Datasets **8**
3.1 Dataset 1 8
3.2 Dataset 2 8
3.3 Dataset 3 9
3.4 Dataset 4 9
3.5 Dataset 5 10

4 Codes **11**
4.1 Code 1 11
4.2 Code 2 12

5 Offset Correction **12**

6 Detection Performance Analysis **13**
6.1 Single Class: Spots 13
6.2 Single Class: Ring-Shaped 14
6.3 Two Classes: Spots + Ring-Shaped 15

7 Experimental Setup **15**

8 Sample Preparation **16**

9 Orientation Detection (YOLOTrack 1.1) **16**

10 Video Files **17**

1 Neural Network Design

1.1 Architecture

We adapted the "Tiny" version of the YOLOv2 architecture: TinyYOLOv2 [1]. It uses 9 convolutional layers with a 3×3 kernel and 6 max-pooling layers with a 2×2 kernel (Fig. S1, Tab. S1) to simultaneously predict multiple bounding boxes and class probabilities for those boxes. The last convolutional layer has a 1×1 kernel and reduces the data to the output shape $13 \times 13 \times B \cdot (4 + 1 + C)$.

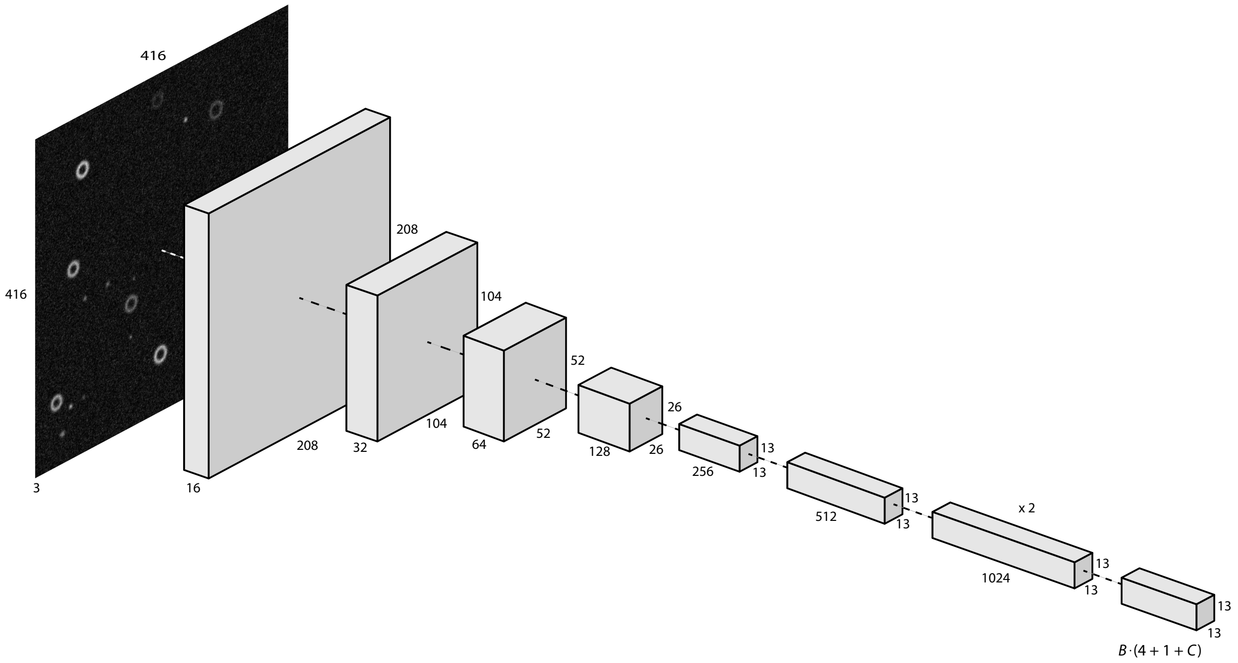

Figure S1: Illustation of the TinyYOLOv2 architecture.

Table S1: TinyYOLOv2 architecture.

Type	Filters	Size/Stride	Output
Input			416×416
Conv.	16	$3 \times 3/1$	416×416
Maxpool		$2 \times 2/2$	208×208
Conv.	32	$3 \times 3/1$	208×208
Maxpool		$2 \times 2/2$	104×104
Conv.	64	$3 \times 3/1$	104×104
Maxpool		$2 \times 2/2$	52×52
Conv.	128	$3 \times 3/1$	52×52
Maxpool		$2 \times 2/2$	26×26
Conv.	256	$3 \times 3/1$	26×26
Maxpool		$2 \times 2/2$	13×13
Conv.	512	$3 \times 3/1$	13×13
Maxpool		$2 \times 2/1$	13×13
Conv.	1024	$3 \times 3/1$	13×13
Conv.	1024	$3 \times 3/1$	13×13
Conv.	$(4 + 1 + C) \cdot B$	$1 \times 1/1$	13×13
Output			

1.2 Output Decoding

The network takes an input RGB image of the size 416×416 pixels and divides that input image into 13×13 grid cells (Fig. 1a). For each grid cell it predicts B bounding boxes and for each bounding box an object confidence as well as probabilities for each of the C classes. The output is encoded in a $13 \times 13 \times B \cdot (4 + 1 + C)$ tensor. Since there are $13 \times 13 = 169$ grid cells, for $B = 5$, the network predicts $169 \cdot 5 = 845$ bounding boxes at once (Fig. 1b). For $C = 2$ classes each bounding box is then described by $4 + 1 + C = 7$ data elements (Fig. S2):

- t_x, t_y, t_w, t_h of the bounding box
- object confidence p_0
- probabilities for all classes $(p_1 \dots p_C)$

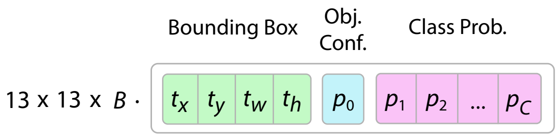

Figure S2: The output shape of the YOLOv2 network.

YOLOv2 predicts the coordinates of the bounding boxes relative to the location of the grid cell using anchor boxes. This bounds the ground truth to fall between 0 and 1. If the location of the grid cell is c_x, c_y and the relative width and height of the anchor box are b_w, b_h, the predictions t_x, t_y, t_w, t_h correspond to:

$$
\begin{aligned}
x &= \sigma(t_x) + c_x \ , \\
y &= \sigma(t_y) + c_y \ , \\
w &= b_w \, \mathrm{e}^{t_w} \ , \\
h &= b_h \, \mathrm{e}^{t_h} \ ,
\end{aligned}
$$

where σ is the logistic activation function, x, y the center location and w, h the width and height of the bounding box relative to the input image size. The anchor boxes are a set of $B = 5$ predefined bounding boxes of a certain height and width. These boxes are defined to capture the aspect ratio of specific object classes. The network does not directly predict bounding boxes, but rather predicts the probabilities and refinements that correspond to a set of anchor boxes.
The object confidence p_0 for the bounding box is the probability that the bounding box contains an object whereas $p_1, p_2, \dots, p_C$ are the probabilities that the object belongs to a certain class. From the 845 predicted bounding boxes most will have a very low object confidence, so we only keep the boxes whose object confidence is larger than a certain object threshold (Fig. 1c). There still may be boxes remaining that largely overlap with each other but belong to the same object (Fig. S3a).

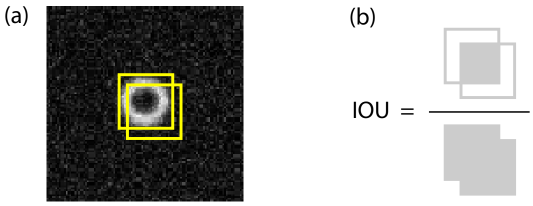

Figure S3: (a) Example of duplicate bounding boxes before the non-maximum suppression (NMS). (b) The intersection over union (IOU) of the two bounding boxes.

To remove these duplicate bounding boxes a non-maximum suppression (NMS) algorithm is applied. The algorithm selects the bounding box with the highest confidence and removes any bounding boxes with an intersection over union (Fig. S3b) larger than a given NMS threshold. Finally, the class of the bounding box is assigned according to the highest class probability.

2 Neural Network Training

The network is trained using a sum-squared error between the predictions and the ground truth to calculate the loss (Section 2.1). We trained the network with synthetic images (Section 2.2) with a size of 416×416 pixels. Whereas we trained the network with grayscale images it also possible to train the network with colored images. For each image the ground truth for the bounding boxes and the classes are stored in an associated XML file in the Pascal VOC format. A dataset comprises a set of training images, a set of validation images and a set of test images. Typically, the size of the validation set is 20 % of the size of the training set. We trained the network without pre-trained weights for 10 epochs and batch sizes of 8. For details on the implementation in Python/Keras see Code 1 and Section 4.

2.1 Loss Function

YOLOv2 uses the following sum-squared error for the loss function [2]:

$$
\begin{aligned}
\text{Loss} \;=\; & \lambda_{\text{loc}} \sum_{i=0}^{G^2} \sum_{j=0}^{B} 1_{ij}^{\text{obj}} \left[\left(x_i - \hat{x}_i\right)^2 + \left(y_i - \hat{y}_i\right)^2 \right] \\
& + \lambda_{\text{loc}} \sum_{i=0}^{G^2} \sum_{j=0}^{B} 1_{ij}^{\text{obj}} \left[\left((w_i)^{\frac{1}{2}} - (\hat{w}_i)^{\frac{1}{2}}\right)^2 + \left((h_i)^{\frac{1}{2}} - (\hat{h}_i)^{\frac{1}{2}}\right)^2 \right] \\
& + \sum_{i=0}^{G^2} \sum_{j=0}^{B} 1_{ij}^{\text{obj}} \left(C_i - \hat{C}_i\right)^2 \\
& + \lambda_{\text{no obj}} \sum_{i=0}^{G^2} \sum_{j=0}^{B} 1_{ij}^{\text{no obj}} \left(C_i - \hat{C}_i\right)^2 \\
& + \sum_{i=0}^{G^2} \sum_{j=0}^{B} 1_{i}^{\text{obj}} \sum_{c \in \text{classes}} \left(p_i(c) - \hat{p}_i(c)\right)^2 .
\end{aligned}
$$

The first two terms represent the localization loss, terms 3 and 4 the confidence loss and the last term the classification loss.

General Remarks

(a) The loss function penalizes classification errors only if an object is present in that grid cell.

(b) Since there are $B = 5$ bounding boxes for each cell we need to choose one of them for the loss. This will be the box with the highest IOU with the ground truth box so the loss will penalize the localization loss if that box is responsible for the ground truth box.

(c) The sum-squared error weights localization errors equally with classification errors.

1st Term

$$
\lambda_{\text{loc}} \sum_{i=0}^{G^2} \sum_{j=0}^{B} 1_{ij}^{\text{obj}} \left[\left(x_i - \hat{x}_i\right)^2 + \left(y_i - \hat{y}_i\right)^2 \right]
$$

SSE between the predicted box location (x, y) and the ground truth location $(\hat{x}, \hat{y})$. We sum over all 13×13 grid cells ($G = 13$) and for each grid cell we sum over all 5 boxes ($B = 5$).

To comply with (a) and (b) a binary variable 1_{ij}^{obj} is used so that $1_{ij}^{\text{obj}} = 1$ if box j in grid i contains an object **and** box j is responsible of detecting that object, otherwise 0 (The box is responsible for detecting an object if it has the highest IOU with the ground truth box between the B boxes).

As mentioned in (c) the SSE weights localization errors equally with classification errors. To give the localization error a higher weight in the loss function the parameter $\lambda_{\text{loc}} = 5$ is used.

2nd Term

$$\lambda_{\text{loc}} \sum_{i=0}^{G^2} \sum_{j=0}^{B} 1_{ij}^{\text{obj}} \left[\left((w_i)^{\frac{1}{2}} - (\hat{w}_i)^{\frac{1}{2}} \right)^2 + \left((h_i)^{\frac{1}{2}} - (\hat{h}_i)^{\frac{1}{2}} \right)^2 \right]$$

The 2nd term is similar to the 1st term, but calculates the SSE in the box dimensions. To reflect that small deviations in large boxes matter less that in small boxes YOLO uses the square root of w and h. Otherwise the SSE would weight errors in large boxes and small boxes equally.

3rd Term

$$\sum_{i=0}^{G^2} \sum_{j=0}^{B} 1_{ij}^{\text{obj}} \left(C_i - \hat{C}_i \right)^2$$

This is the confidence error where $0 \leq C \leq 1$ and $\hat{C} = 1$.

4th Term

$$\lambda_{\text{no obj}} \sum_{i=0}^{G^2} \sum_{j=0}^{B} 1_{ij}^{\text{no obj}} \left(C_i - \hat{C}_i \right)^2$$

If there is no object in the grid we only need to take care about the confidence C (The confidence needs to be zero when there is no object). The variable $1_{ij}^{\text{no obj}} = 1$ if box j in grid i contains no object **or** box j is not responsible of detecting that object, otherwise 0.

Since many grid cells do not contain any object, this pushes the confidence scores of those cells quickly towards zero This can lead the training to diverge early. To attenuate the decrease of the loss from confidence predictions of boxes that do not contain objects the parameter $\lambda_{\text{no obj}} = 0.5$ is used.

5th Term

$$\sum_{i=0}^{G^2} \sum_{j=0}^{B} 1_{i}^{\text{obj}} \sum_{c \,\in\, \text{classes}} \left(p_i(c) - \hat{p}_i(c) \right)^2$$

Here, the errors for all class probabilities in the 13×13 grid cells are summed.

2.2 Synthetic Image Generation

Here we list the generation functions used to simulate the darkfield images of nano- and microparticles, Janus-type as well as rod-like and elliptical microparticles. Note that all images are assumed to be in focus.

2.2.1 Spots

Function used to generate a Gaussian intensity distribution:

$$I(x, y, \{x_0, y_0; \sigma\}) = I_0 \exp\left(-\frac{(x-x_0)^2 + (y-y_0)^2}{2\sigma^2}\right) \tag{1}$$

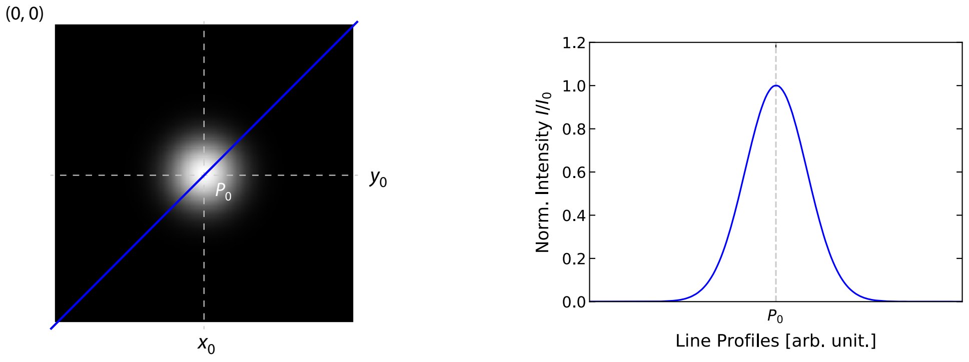

Figure S4: Example of an intensity distribution generated with Eqn. (1).

2.2.2 Ring-Shaped

Function used to generate a ring-shaped intensity distribution:

$$I(x, y, \{x_0, y_0; \sigma, R\}) = I_0 \exp\left(-\frac{\left(\sqrt{(x-x_0)^2 + (y-y_0)^2} - R\right)^2}{2\sigma^2}\right) \tag{2}$$

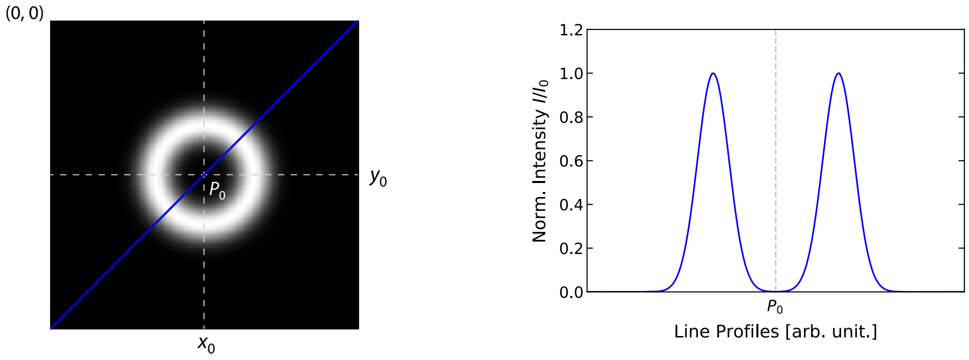

Figure S5: Example of a ring-shaped intensity distribution generated with Eqn. (2).

2.2.3 Janus-Type

Function used to generate a Janus-type intensity distribution:

$$I(x, y, \{x_0, y_0, \varphi; \sigma, R\}) = I_0 \cos^2(\alpha) \exp\left(-\frac{\left(\sqrt{(x-x_0)^2 + (y-y_0)^2} - R\right)^2}{2\sigma^2}\right) , \tag{3}$$

Here, α is given as:

$$\alpha = \frac{1}{2} \arccos \left(\frac{(x' - x_0)}{\sqrt{(x' - x_0)^2 - (y' - y_0)^2}} \right) \ ,$$

where x' and y' are coordinates in the reference frame of the particle center:

$$x' = (x - x_0)\ \cos(\varphi) - (y - y_0)\ \sin(\varphi)\ ,$$
$$y' = (x - x_0)\ \sin(\varphi) + (y - y_0)\ \cos(\varphi)\ ,$$

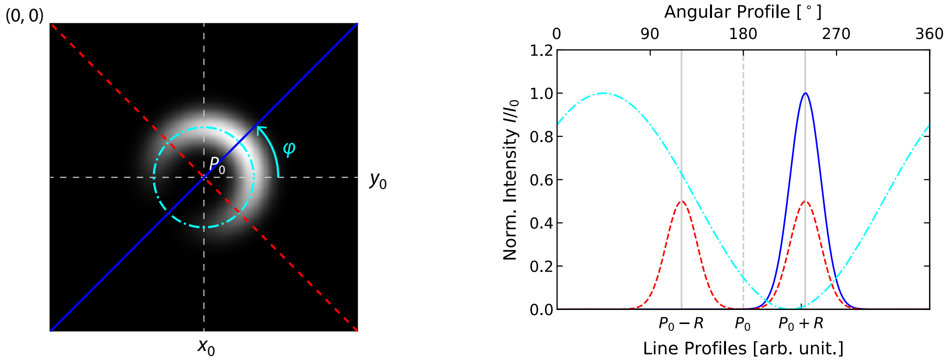

Figure S6: Example of a Janus-type intensity distribution generated with Eqn. (3).

2.2.4 Ellipse

Function used to generate an elliptical, two-dimensional Gaussian intensity distribution:

$$I(x, y, \{x_0, y_0, \varphi; \sigma_x, \sigma_y\}) = I_0 \exp\left(-(a(x - x_0)^2 + 2b(x - x_0)(y - y_0) + c(y - y_0)^2)\right) \ , \qquad (4)$$

where a, b and c are given in terms of the orientation angle θ:

$$a = \frac{\cos(\varphi)^2}{2\sigma_x^2} + \frac{\sin(\varphi)^2}{2\sigma_y^2} \ , \quad b = -\frac{\sin(2\varphi)}{4\sigma_x^2} + \frac{\sin(2\varphi)}{4\sigma_y^2} \ , \quad c = \frac{\sin(\varphi)2}{2\sigma_x^2} + \frac{\cos(\varphi)^2}{2\sigma_y^2} \ .$$

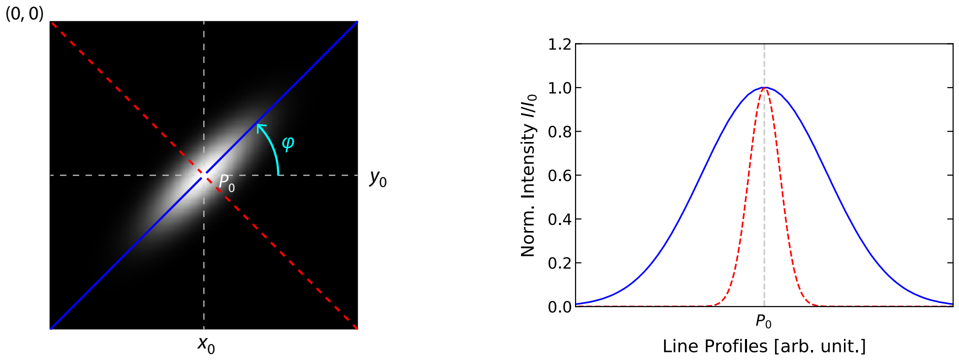

Figure S7: Example of an elliptical intensity distribution generated with Eqn. (4).

2.2.5 Rod-Like

Function used to generate a rod-like intensity distribution:

$$I(x, y, \{x_0, y_0, \varphi; \ell, w, \sigma\}) = I_0 \{\Pi(x_0, y_0, \varphi; \ell, w) * g(\sigma)\}(x, y) \qquad (5)$$

Here, $*$ denotes the convolution of a two-dimensional, rectangular function Π with a Gaussian function g, where ℓ, w are the length and width of the rectangle and σ^2 the variance of the Gaussian function.

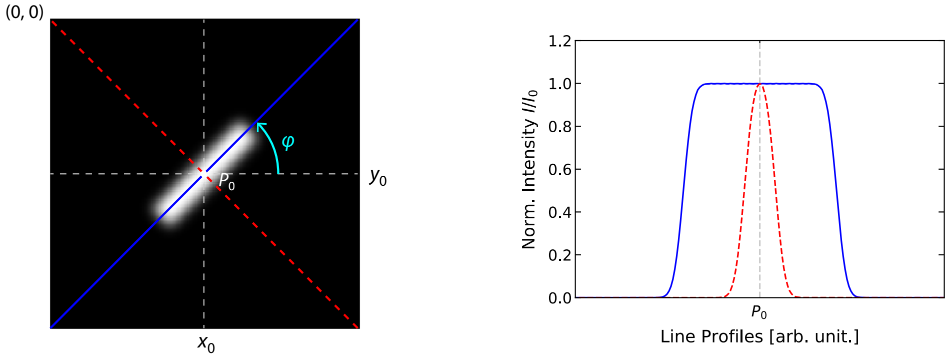

Figure S8: Example of a rod-like intensity distribution generated with Eqn. (5).

3 Datasets

This section gives an overview of the investigated synthetic datasets and the parameters used for their generation. Here, [a ... b] denotes a uniform distribution of random samples between a and b. The SNR of an image is defined as the ratio of the average signal value to the standard deviation of the signal.

3.1 Dataset 1

Dataset 1 comprises 25000 images for training and 5000 images for validation. The SNR of the images is randomly distributed with SNR $= [1 \dots 30]$.

Table S2: Parameters for Dataset 1.

Particle Type	Eqn.	Parameters	# Particles
Spots	(1)	$I_0 = [0.1 \dots 1]$<br>$\sigma = 3$	$N_1 = [1 \dots 20]$

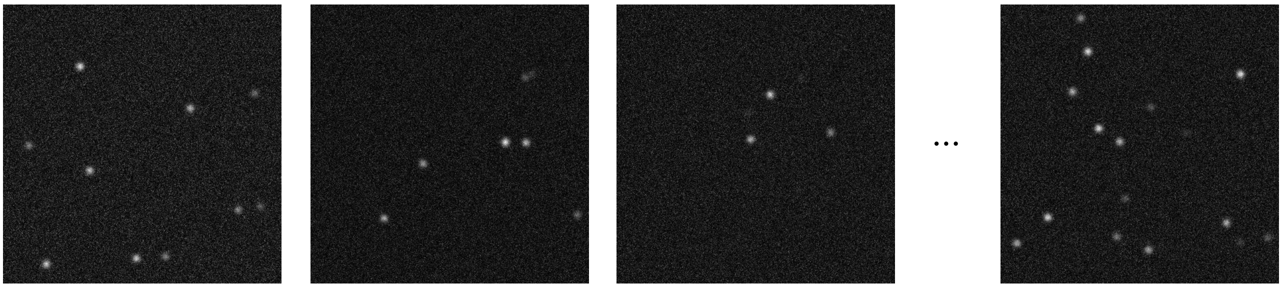

Figure S9: Sample images from Dataset 1.

3.2 Dataset 2

Dataset 2 comprises 25000 images for training and 5000 images for validation. The SNR of the images is randomly distributed with SNR $= [1 \dots 30]$.

Table S3: Parameters for Dataset 2.

**Particle Type**	**Eqn.**	**Parameters**	**# Particles**
Ring-Shaped	(2)	$I_0 = [0.1 \ldots 1]$<br>$R = 8$<br>$\sigma = 4$	$N_2 = [1 \ldots 10]$

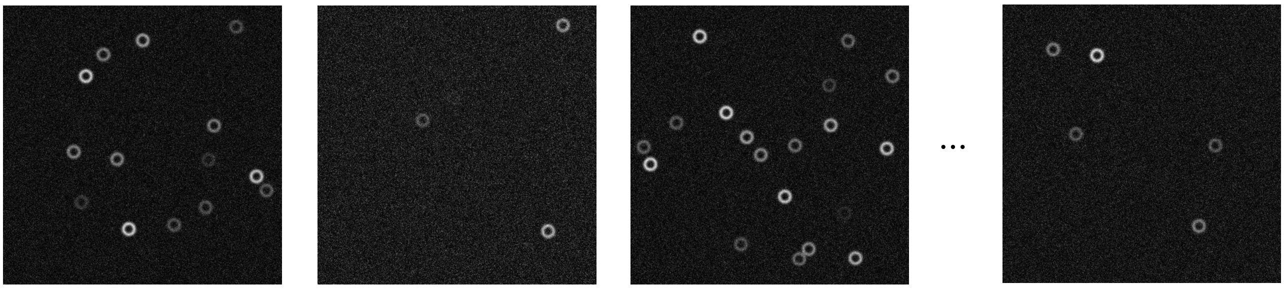

Figure S10: Sample images from Dataset 2.

3.3 Dataset 3

Dataset 3 comprises 25000 images for training and 5000 images for validation. The SNR of the images is randomly distributed with SNR = $[1 \ldots 30]$.

Table S4: Parameters for Dataset 3.

**Particle Type**	**Eqn.**	**Parameters**	**# Particles**
Spot	(1)	$I_0 = [0.1 \ldots 1]$<br>$\sigma = 3$	$N_1 = [1 \ldots 20]$
Ring-Shaped	(2)	$I_0 = [0.1 \ldots 1]$<br>$R = 8$<br>$\sigma = 4$	$N_2 = [1 \ldots 10]$

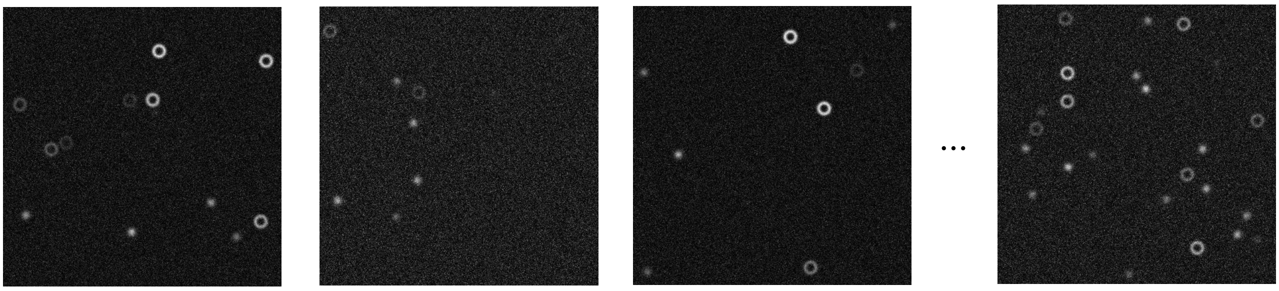

Figure S11: Sample images from Dataset 3.

3.4 Dataset 4

Dataset 4 comprises 25000 images for training and 5000 images for validation. The SNR of the images is randomly distributed with SNR = $[1 \ldots 30]$.

Table S5: Parameters for Dataset 4.

**Particle Type**	**Eqn.**	**Parameters**	**# Particles**
Spot	(1)	$I_0 = [0.1, \ldots 1]$<br>$\sigma = [2 \ldots 4]$	$N_1 = [1 \ldots 20]$
Ring-Shaped	(2)	$I_0 = [0.1 \ldots 1]$<br>$R = [6 \ldots 10]$<br>$\sigma = 4$	$N_2 = [1 \ldots 10]$

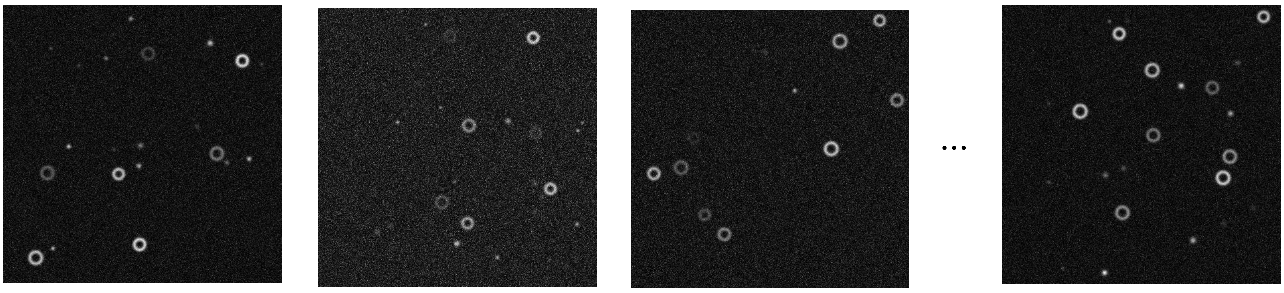

Figure S12: Sample images from Dataset 4.

3.5 Dataset 5

Dataset 5 comprises 125000 images for training and 25000 images for validation. The SNR of the images is randomly distributed with SNR = $[1 \ldots 30]$.

Table S6: Parameters for Dataset 5.

**Particle Type**	**Eqn.**	**Parameters**	**# Particles**
Spot	(1)	$I_0 = [0.1, \ldots 1]$<br>$\sigma = [2 \ldots 4]$	$N_1 = [5 \ldots 15]$
Ring-Shaped	(2)	$I_0 = [0.1 \ldots 1]$<br>$R = [6 \ldots 10]$<br>$\sigma = 2$	$N_2 = [1 \ldots 5]$
Janus-Type	(3)	$I_0 = [0.1 \ldots 1]$<br>$R = 8$<br>$\sigma = 3$	$N_3 = [1 \ldots 5]$
Ellipses	(4)	$I_0 = [0.1 \ldots 1]$<br>$\sigma_x = 4$<br>$\sigma_y = 12$	$N_4 = [1 \ldots 5]$
Rods	(5)	$I_0 = [0.1 \ldots 1]$<br>$w = 3$<br>$\ell = [15 \ldots 30]$<br>$\sigma = 2$	$N_5 = [1 \ldots 5]$

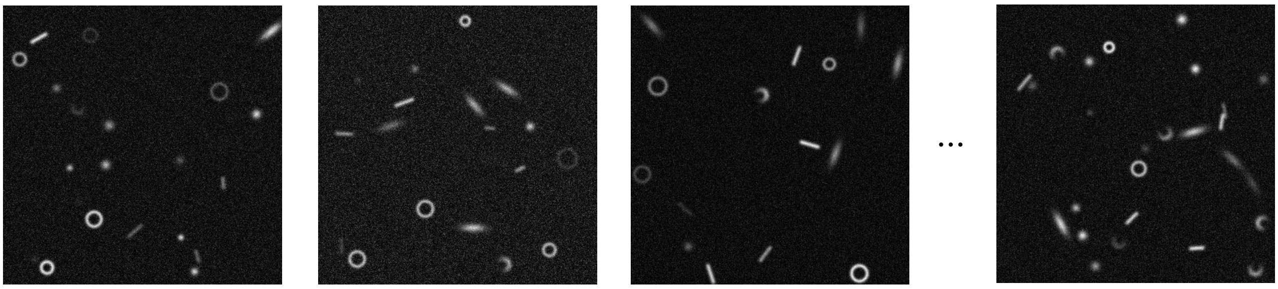

Figure S13: Sample images from Dataset 5.

4 Codes

The network is trained and evaluated in Python/Keras using the TensorFlow backend [3–5]. For real-time inference the model graph is exported as protocol buffer file (`*.pb`) and parameters required to decode the YOLOTrack 1.0 output are exported to an INI file (`*.ini`). These files are imported by the C based dynamic link libraries `TF.dll`, `YOLOTrack10.dll` that are easily integrable in other programming languages such as LabVIEW and C++. Fig. S14 shows the structure of the software framework:

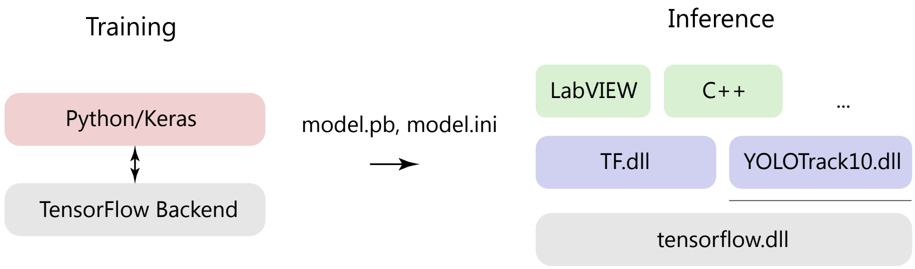

Figure S14: The structure of the developed framework used for training and GPU supported real-time detection.

The `TF.dll` is a general TensorFlow library for model inference build on top of the TensorFlow C API (`tensorflow.dll`). It can be used with any TensorFlow model and is not specific to YOLOTrack. The `YOLOTrack10.dll` adds specific functions required to decode the YOLOTrack 1.0 output and does not depend on the TensorFlow C API.

4.1 Code 1

Code 1 contains the YOLOTrack 1.0 implementation. The code repository is structured in the following directories:

- **YOLOTrack 1.0 Training (Python)**: Jupyter notebooks and Python modules for synthetic image generation, training and exporting YOLOTrack 1.0 models with Python/Keras using the TensorFlow backend
- **TF DLL**: DLL for running TensorFlow models on GPU (`TF.dll`)
- **YOLOTrack 1.0 DLL**: DLL for decoding the YOLOTrack 1.0 output tensor (`YOLOTrack10.dll`)
- **YOLOTrack 1.0 Inference (LabVIEW)**: Example for importing and running a trained YOLOTrack 1.0 model with LabVIEW
- **YOLOTrack 1.0 Inference (C++)**: Example for importing and running a trained model with Qt/C++

The system requirements and a detailed documentation can be found in the `README.md` files contained in each directory. A maintained version of Code 1 can be downloaded at:

https://github.com/Molecular-Nanophotonics/YOLOTrack-1.0

4.2 Code 2

Code 2 contains an extended version of YOLOTrack, called YOLOTrack 1.1, to allow for the detection of oriented bounding boxes (see Section 9 for details). The repository structure is largely similar to Code 1 and structured as follows:

- **YOLOTrack 1.1 Training (Python)**: Jupyter notebooks and Python modules for synthetic image generation, training and exporting YOLOTrack 1.1 models with Python/Keras using the TensorFlow backend.
- **TF DLL**: DLL for running TensorFlow models on GPU (`TF.dll`)
- **YOLOTrack 1.1 DLL**: DLL for decoding the YOLOTrack 1.1 output tensor (`YOLOTrack11.dll`)
- **YOLOTrack 1.1 Inference (LabVIEW)**: Example for importing and running a trained YOLOTrack 1.1 model with LabVIEW
- **YOLOTrack 1.1 Inference (C++)**: Example for importing and running a trained model with Qt/C++

The **TF DLL** directory is the same as in Code 1 and just added for convenience. The system requirements and the documentation can be found in the `README.md` files contained in each directory. A maintained version of Code 2 can be downloaded at:

https://github.com/Molecular-Nanophotonics/YOLOTrack-1.1

5 Offset Correction

The trained network can have a sub-pixel localization offset. However, once trained the offset vector is constant and can be corrected by subtraction (Fig. S15). The offset vector is independent on the SNR but depends on the object class. In general, a correction is only required when seeking for sub-pixel resolution.

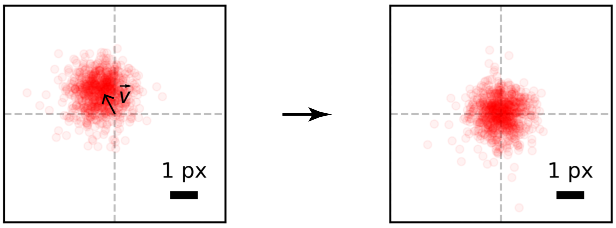

Figure S15: Example for the correction of an offset vector $\boldsymbol{v}$.

To correct the offset for a specific class the error/offset from the ground truth is sampled with 1000 synthetic images containing only one object of that class at randomized positions. The offset vector is then calculated from the mean of the offset distribution. The offset correction has to be done for every model and individually for all object classes.

6 Detection Performance Analysis

6.1 Single Class: Spots

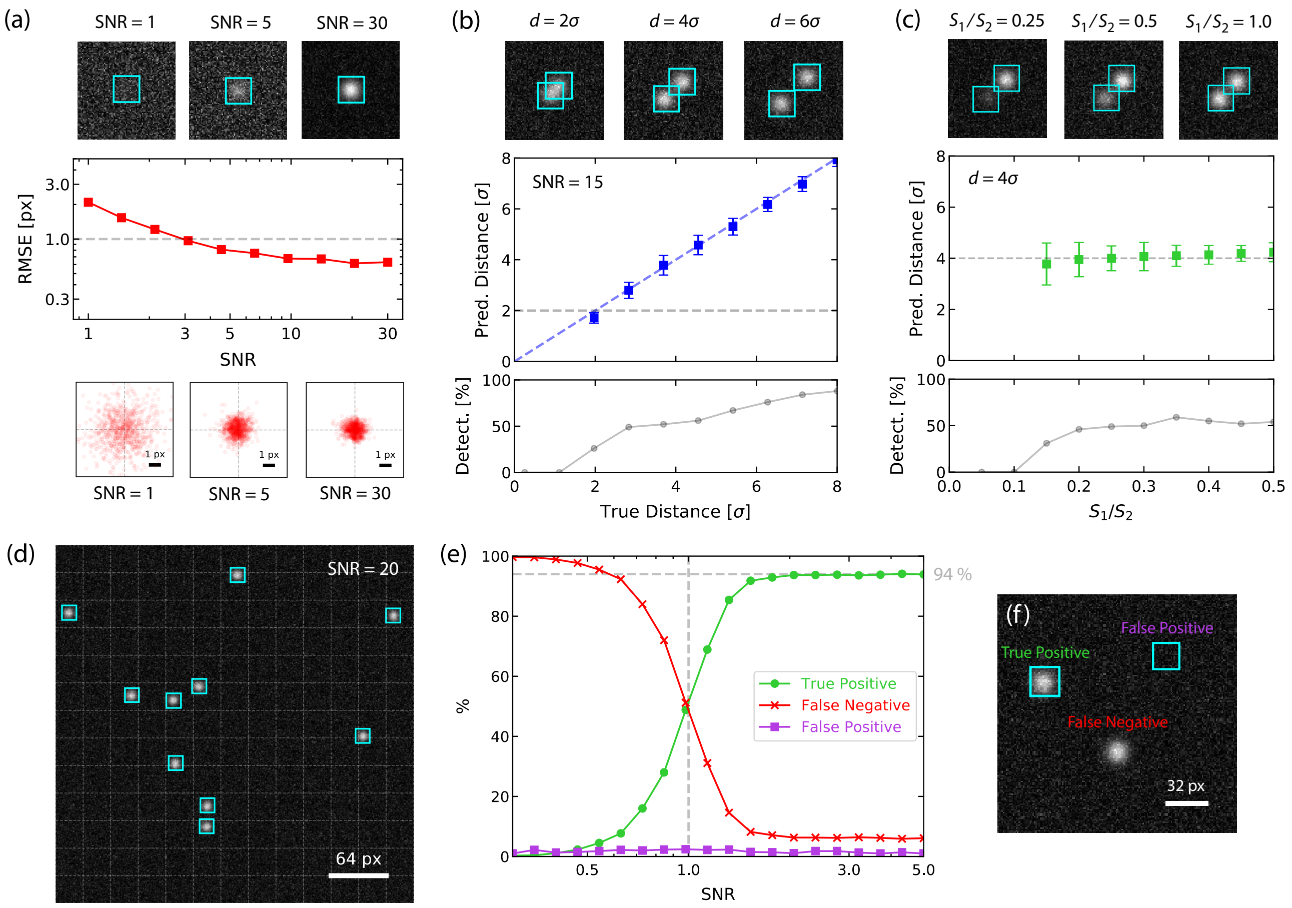

Figure S16: Single Class Training Set (Dataset 1, Section 3.1). (a) Root mean-squared error (RMSE), i.e., the root mean-squared distance between the true and the predicted center position, as function of the signal-to-noise ratio (SNR). Each SNR value was sampled with $S = 1000$ images containing $N = 1$ particle with randomized position. (b) Predicted distance as function of the true distance for SNR = 15. Each distance was sampled with $S = 100$ images containing $N = 2$ particles with randomized position and orientation. The error bars indicate the standard deviation of the predicted distances. The lower graph depicts the percentage of images where two particles have been detected. (c) Predicted distance as function of the signal ratio averaged over $S = 100$ sample images containing $N = 2$ particles with randomized position and orientation. The error bars indicate the standard deviation of the predicted distances. The lower graph, again, depicts the percentage of images where two particles have been detected. (d) The detection output for an image with $N = 10$ particles and SNR = 20. (e) Percentage of true positives (green dots), false negative (red crosses) and false positive (violet squares) detection as function of the SNR. Each SNR value was a sample with $S = 100$ images containing $N = 10$ particles at randomized positions as plotted in (d). (f) A visualization of true positive, false negative and false positive detections. For all images the detection output was decoded with an object threshold of 0.6 and a NMS threshold of 0.45

6.2 Single Class: Ring-Shaped

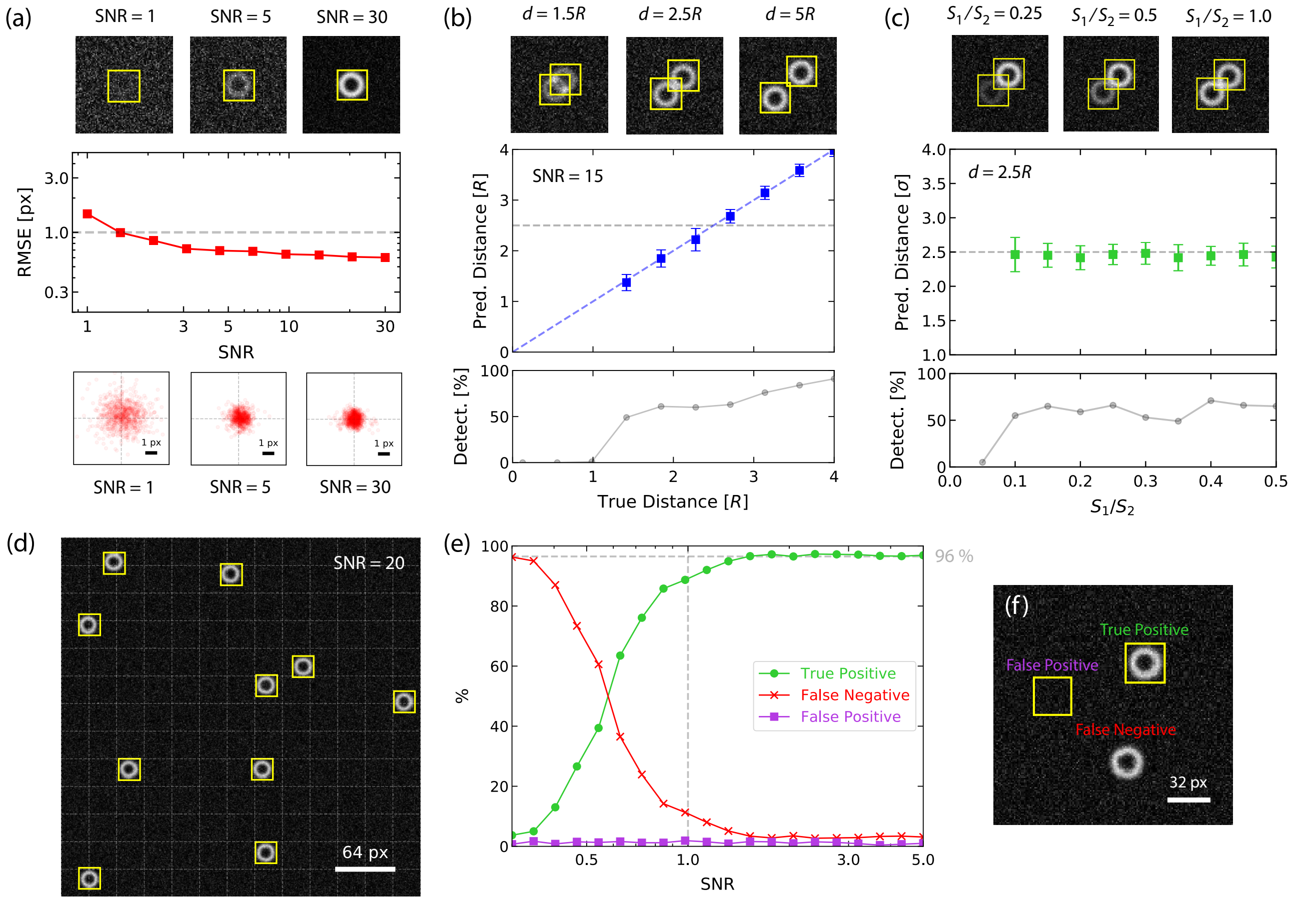

Figure S17: Single Class Training Set (Dataset 2, Section 3.2). (a) Root mean-squared error (RMSE), i.e., the root mean-squared distance between the true and the predicted center position, as function of the signal-to-noise ratio (SNR). Each SNR value was sampled with $S = 1000$ images containing $N = 1$ particle with randomized position. (b) Predicted distance as function of the true distance for SNR $= 15$. Each distance was sampled with $S = 100$ images containing $N = 2$ particles with randomized position and orientation. The error bars indicate the standard deviation of the predicted distances. The lower graph shows the percentage of images where two particles have been detected. (c) Predicted distance as function of the intensity ratio averaged over $S = 100$ sample images containing $N = 2$ particle randomized position and orientation. The error bars indicate the standard deviation of the predicted distances. The lower graph shows the percentage of images where two particles have been detected. (d) The detection output for an image with $N = 10$ particles and SNR $= 20$. (e) Percentage of true positives (green dots), false negative (red crosses) and false positive (violet squares) detection as function of the SNR. Each SNR value was sample with $S = 100$ images containing $N = 10$ particles at randomized positions as shown in (d). (f) A visualization of true positive, false negative and false positive detections. For all images the detection output was decoded with an object threshold of 0.6 and a NMS threshold of 0.45

6.3 Two Classes: Spots + Ring-Shaped

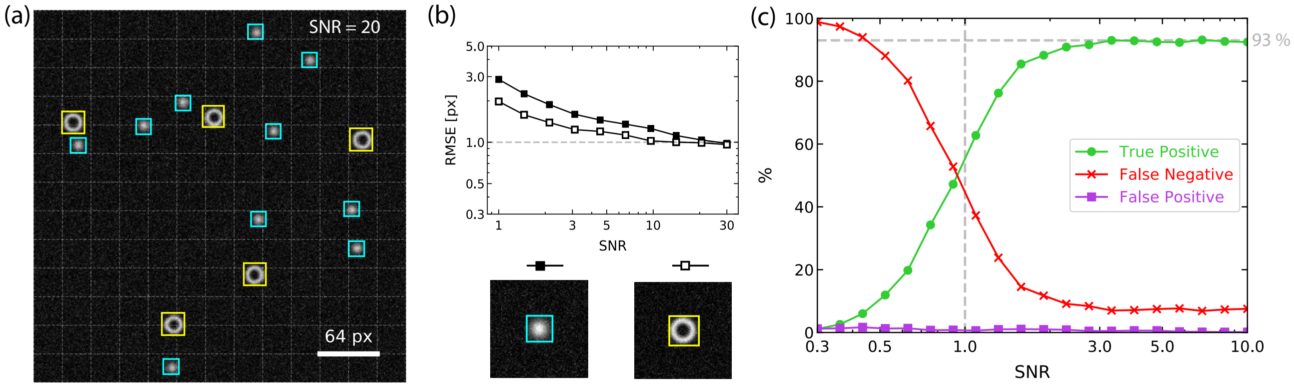

Figure S18: Two training classes. (a) The predicted particle locations and classes for a test image with $N_1 = 10$ spots and $N_2 = 5$ ring-shaped particles at a signal-to-noise ration (SNR) of 20. (b) The root mean-squared error of the localization for both particle classes as function of the SNR. Each class and SNR value was sampled with $S = 1000$ images containing $N = 1$ particle with randomized position. (c) Percentage of true positive (green dots), false negative (red crosses) and false positive (violet squares) detections as function of the SNR. Each SNR value was sampled with $S = 100$ test images containing $N_1 = 10$ spots and $N_1 = 5$ ring-shaped particles at randomized positions as illustrated in (a).

7 Experimental Setup

A sketch of the experimental setup is shown in Fig. S19. The experimental setup consists of an inverted microscope (Olympus, IX71) with a mounted piezo translation stage (Physik Instrumente, P-733.3). The microparticles are heated by a focused, continuous wave laser at a wavelength of 532 nm (CNI, MGL-III-532). The beam diameter is increased by a beam expander and sent to an acousto-optic deflector (AA Opto-Electronic, DTSXY-400-532) and a lens system to steer the laser focus in the sample plane. The deflected beam is focused by an oil-immersion objective (Olympus, UPlanApo ×100/1.35, Oil, Iris, NA 0.5 - 1.35) to the sample plane ($w_0 \approx 1$ µm beam waist in the sample plane).

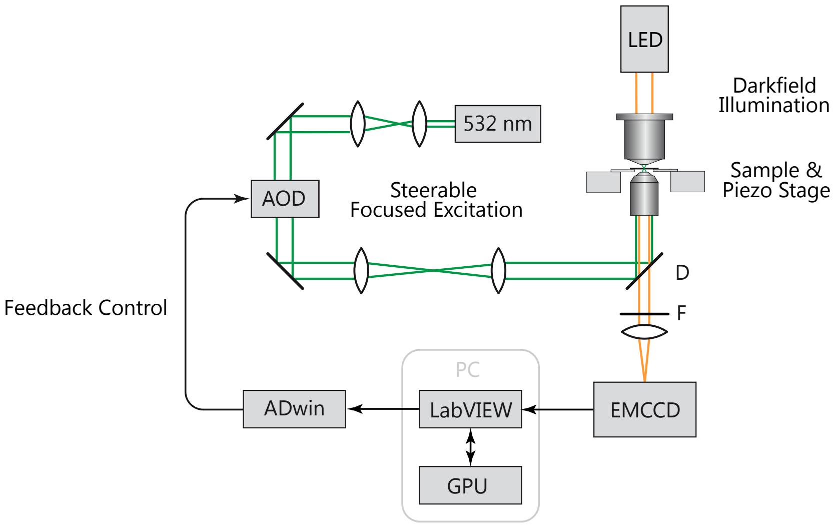

Figure S19: Sketch of the setup used for experiments.

The sample is illuminated with an oil-immersion darkfield condenser (Olympus, U-DCW, NA 1.2 - 1.4) and a white-light LED (Thorlabs, SOLIS-3C). The scattered light is imaged by the objective and a tube lens (250 mm) to an EMCCD (electron-multiplying charge-coupled device) camera (Andor, iXon DV885LC). The variable numerical aperture of the objective was set to a value below the minimum aperture of the darkfield condenser. The dichroic beam splitter (D) was selected to reflect the laser wavelength (Omega Optical, 560DRLP) and a notch filter (F) is used to block any remaining back reflections from the laser (Thorlabs, NF533-17). The acousto-optic deflector (AOD), as well as the piezo stage, are driven by an AD/DA (analog-digital/digital-analog) converter (Jäger Messtechnik, ADwin-Gold II). A LabVIEW program running on a desktop PC (Intel Core i7 2600 4 $\times$ 3.40 GHz CPU) is used to record and process the images as well as to control the AOD feedback via the AD/DA converter. To get the fastest possible image processing the LabVIEW program is interfacing a GeForce GTX 1660 Ti GPU to run the model inference at a frame rate of up to 100 fps.

8 Sample Preparation

The sample consists of two glass cover slips (22 mm $\times$ 22 mm) confining a thin liquid film. First, the cover slips were thoroughly cleaned by rinsing successively with acetone, isopropyl and Milli-Q water and dried with a nitrogen gun. To prevent sticking of the microparticles, the glass surfaces were passivated with Pluronic F-127 (Sigma-Aldrich). To attain the adsorption of Pluronic F-127 in a brush-like configuration the cleaned, hydrophilic cover slips were rendered hydrophobic with a thin layer of polystyrene (PSS-Polymer, $M_\mathrm{w} = 88$ kDa, PDI = 1.66). Therefore, 30 µl of 2 % polystyrene in toluene was spin-coated at 8,000 rpm onto the cover slips, resulting in a polystyrene layer thickness of about 100 nm. Subsequently, the cover slips are immersed in 1 % Pluronic F-127 solution for 10 min. Thereafter, the cover slips were briefly dipped in Milli-Q water and dried with a nitrogen gun. Subsequently, the edges of one cover slip was covered with a thin layer of PDMS (polydimethylsiloxane) for sealing. The particle solution used for the experiments was prepared by mixing 2.2 µm diameter gold-coated melamine formaldehyde (MF) particles (MicroParticles) and 0.5 µm diameter polystyrene particles (Polyscience) in 0.1 % Pluronic F-127 solution. The surface of the MF particles is uniformly coated with gold nanoparticles of about 10 nm diameter with a surface coverage of about 10 %. To passivate the surface of the particles they have been washed in a 1 % Pluronic F-127 solution before mixing. Finally, 0.5 µl of the mixed particle suspension is pipetted in the middle of one of the cover slips and the other is placed on top. Depending on the area covered by the liquid, typically about 18 mm $\times$ 18 mm, the resulting liquid film height is about 3 µm.

9 Orientation Detection (YOLOTrack 1.1)

To allow for an orientation detection the network needs to be modified. We normalized the 360° range of possible orientations to a range from 0 to 1 and adapted YOLOTrack 1.0 to directly predict the orientation of the bounding box via a single regression number φ (Fig. S20).

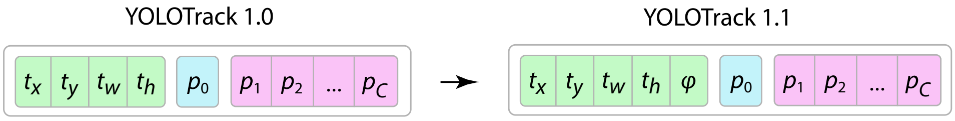

Figure S20: The output shape of YOLOTrack 1.0 vs. YOLOTrack 1.1.

In the loss function the mean squared error between the ground truth $\hat{\varphi}_i$ and the predicted angle φ_i was added:

$$\sum_{i=0}^{G^2} \sum_{j=0}^{B} 1_{ij}^{\mathrm{obj}} \left(\varphi_i - \hat{\varphi}_i\right)^2 \ .$$

To bound the output between 0 and 1 the logistic activation function σ was used. The annotation XML file format was adapted by adding a `<orientation></orientation>` tag, requiring also to adapt the annotation parsing and the batch generation. The implementations details can be found in Code 2.

10 Video Files

Video 1: Single particle driven between two target positions.

Video 2: Single particle confined at a target position.

Video 3: Control of 6 active particles in a hexagon with a background of passive particles.

Video 4: Control of 6 active particles at low SNR with in a grid pattern.

Video 5: Control of 9 active particles at low SNR in a grid pattern that is transformed in its size.

Video 6: Control of 9 active particles at low SNR that are transformed from a grid to circular pattern.

References

[1] J. Redmon and A. Farhadi, YOLO9000: Better, Faster, Stronger, *arXiv:1612.08242* (2016).

[2] J. Hui, Real-Time Object Detection with YOLO, YOLOv2 and now YOLOv3, *https://medium.com* (2018).

[3] M. Abadi, A. Agarwal, P. Barham, E. Brevdo, Z. Chen *et al.*, TensorFlow: Large-Scale Machine Learning on Heterogeneous Distributed Systems, *arXiv:1603.04467* (2016).

[4] F. Chollet, Keras, *https://keras.io* (2020).

[5] F. Chollet, *Deep learning with Python* (Manning Publications Co, Shelter Island, New York, 2018).
